# Flavaglines Alleviate Doxorubicin Cardiotoxicity: Implication of Hsp27

**DOI:** 10.1371/journal.pone.0025302

**Published:** 2011-10-31

**Authors:** Yohann Bernard, Nigel Ribeiro, Frédéric Thuaud, Gülen Türkeri, Ronan Dirr, Mounia Boulberdaa, Canan G. Nebigil, Laurent Désaubry

**Affiliations:** 1 Therapeutic Innovation Laboratory, UMR7200, CNRS/Université de Strasbourg, Illkirch, France; 2 Institut de Recherche de l'Ecole de Biotechnologie de Strasbourg, FRE 3211, CNRS/Université de Strasbourg, Illkirch, France; Université Joseph Fourier, France

## Abstract

**Background:**

Despite its effectiveness in the treatment of various cancers, the use of doxorubicin is limited by a potentially fatal cardiomyopathy. Prevention of this cardiotoxicity remains a critical issue in clinical oncology. We hypothesized that flavaglines, a family of natural compounds that display potent neuroprotective effects, may also alleviate doxorubicin-induced cardiotoxicity.

**Methodology/Principal Findings:**

Our *in vitro* data established that a pretreatment with flavaglines significantly increased viability of doxorubicin-injured H9c2 cardiomyocytes as demonstrated by annexin V, TUNEL and active caspase-3 assays. We demonstrated also that phosphorylation of the small heat shock protein Hsp27 is involved in the mechanism by which flavaglines display their cardioprotective effect. Furthermore, knocking-down Hsp27 in H9c2 cardiomyocytes completely reversed this cardioprotection. Administration of our lead compound (FL3) to mice attenuated cardiomyocyte apoptosis and cardiac fibrosis, as reflected by a 50% decrease of mortality.

**Conclusions/Significance:**

These results suggest a prophylactic potential of flavaglines to prevent doxorubicin-induced cardiac toxicity.

## Introduction

Anthracyclines are among the most effective anticancer drugs available with antitumor activity against both hematopoietic and solid tumors [1], [2]. However, their clinical utility is markedly hampered by a major risk of cardiotoxicity that may lead to dilated cardiomyopathy and congestive heart failure [3]–[6]. For example, 36% of adults treated with anthracyclines display cardiac dysfunction and the risk of developing a congestive heart failure reaches 48% for a cumulated dose of 700 mg/m^2^. This cardiotoxicity is even more exacerbated in pediatric and elderly population. In a study on childhood leukemia, 57% of the 115 survivors had echocardiographic abnormalities in heart function [5]. Considering that more than 50% of long-term survivors of childhood cancer were treated with an anthracycline, cardiotoxicity of this class of medicine is a widely prevalent problem that cannot be ignored [6].

While the anticancer properties of anthracyclines is due to the inhibition of topoisomerase II, their cardiotoxicity is caused by other mechanisms that involve apoptosis mediated by calcium overload and iron-catalyzed formation of free radicals.

Flavaglines constitute a family of compounds isolated from medicinal plants of Southeast Asia that display a unique array of pharmacological effects [7], [8]. Recently, these compounds have been reported to reduce MPP-induced mesencephalic cell toxicity in a model of dopaminergic neuronal cell damage [9]. A similar effect was observed *in vivo*, when mice were challenged with the neurotoxin MPTP, as a model of Parkinson's disease [9]. This first report of the cytoprotective potential of flavaglines prompted us to examine whether these compounds could have also cardioprotective properties. Indeed, cardiomyocytes and neurons have been shown to share similar mitochondrial-dependent and -independent mechanisms for death and survival [10]–[13].

In this study, we investigated the cardioprotective potential of synthetic flavagline analogs FL1–4 in cellular and animal models of doxorubicin-induced cardiotoxicity. The mechanism by which FL3 leads to cardioprotection was also examined.

## Materials and Methods

### Materials

Flavaglines FL1–4 were synthesized in our laboratory [14]. KRIBB3 and doxorubicin were purchased from Sigma-Aldrich (Saint-Quentin Fallavier, France). Rabbit polyclonal anti-phospho-Hsp27 (Ser 15) antibody, rabbit polyclonal anti-Hsp27 antibody and horseradish peroxidase–conjugated secondary goat anti-rabbit IgG were purchased from Santa Cruz (Santa Cruz Biotechnology, Santa Cruz, CA, USA). Enzyme-linked chemiluminescence for Western blot analyses was purchased from Amersham (Amersham Biosciences, Indianapolis, IN, USA). Hsp27 siRNA antisense and control siRNA, a siRNA sequence not homologous to any known gene, were chemically synthesized by Ambion (Austin, TX, USA). Lipofectamine 2000 was the transfection agent used and was purchased from Invitrogen (Invitrogen, Cergy-Pontoise, France).TdT-mediated dUTP nick end-labeling (TUNEL) assay was purchased from Millipore (Billerica, MA, USA). Male BALB/cByJ mice were purchased from Charles River (Charles River, l'Arbresle, France).

### Cell culture

The H9c2 cardioblast cell line derived from embryonic rat heart was obtained from American Type Culture Collection (Manassas, VA, USA). Cells were grown in Dulbecco's modified Eagle's medium (DMEM) supplemented with 10% fetal calf serum at 37°C in 5% CO**_2_**. The medium was changed every 2–3 days.

### Cell culture and experimental design of in vitro cardiotoxicity assay

H9c2 cells were plated for 24 h in 100 mm Petri dishes at 7×10^3^ cells/cm^2^. Next, the cells were washed and cultured for 12 h in glucose-free medium (Gibco, DMEM w- L-glutamine, w/o D-glucose, sodium pyruvate) supplemented with only 1% fetal calf serum. Cells were then treated with flavaglines FL1–4 or their vehicle for 10 h and then were treated with doxorubicin for an additional 14 h. The doxorubicin concentration and incubation time were chosen as an acute model of cardiotoxicity [15]. KRIBB3 (1 uM) was preincubated for 1 h before flavaglines and treatment in cardiomyocytes. Cells were washed, and subsequently FACS analyses were performed. Unless it is indicated as a chronic model, acute model of doxorubicin-induced cardiotoxicity was used.

### Serum withdrawal-induced cardiotoxicity

H9c2 cells were plated for 24 h in 100 mm Petri dishes at 7×10^3^ cells/cm^2^. Next, the cells were washed and cultured for 12 h in glucose-free medium (Gibco, DMEM w- L-glutamine, w/o D-glucose, sodium pyruvate) supplemented with only 1% fetal calf serum. Cells were then treated with flavagline FL3 (20 nM) or their vehicle for 24, 48 and 72 h. Cells were washed, and subsequently FACS analyses were performed [16].

### FACS analysis

Apoptosis was analyzed by fluorescence-activated cell sorting analysis (FACS-Calibur, Becton-Dickinson Biosciences) [16]. 7×10^3^ Cells were harvested and washed with Annexin Binding Buffer (0.01 M HEPES, 0.14 M NaCl, 2.5 mM CaCl_2_) and labeled with annexin V (dilution 1∶50) and propidium iodide (6.7 µg/ml). All assays were performed at least in triplicate, and the results were analyzed by BD Cell Quest Pro software (Becton-Dickinson Biosciences, Le Pont De Claix, France).

### Western Blotting

H9c2 cells were plated for 24 h on 60 mm Petri dishes at 1.5×10^4^ cells/cm^2^. Next, cells were washed and cultured for 12 h in glucose-free medium (Gibco, DMEM w- L-glutamine, w/o D-glucose, sodium pyruvate) supplemented with only 1% fetal calf serum. Cells were then incubated with FL3 or vehicle for 1 h or 4 h and, after appropriate treatment, washed twice with Phosphate-Buffered Saline (PBS). Cells were harvested with lysis buffer (50 mM Tris-Hcl, 1 mM EDTA, 100 mM NaCl. 0.1% SDS, 1% NP-40, 1 mM Na_3_VO_4_, 1 ug/mL aprotinin, 1 ug/mL pepstatin, 1 ug/mL leupeptin, pH = 7). Whole cell lysates were centrifugated at 12000 g for 15 min at 4°C.The debris were eliminated and 20 µg of protein were separated under denaturing conditions using 12% SDS-PAGE and transferred to a polyvinylidene difluoride membrane. Blots were then incubated with a blocking solution containing 5% fat-free milk powder in PBS plus Tween (0.5% Tween 20) (PBS-T) at room temperature for 1 h. After three washes with PBS-T for 10-min intervals, blots were incubated overnight at 4°C under gentle shacking with respective primary antibody (rabbit polyclonal anti-phospho-Hsp27 (1∶500); rabbit polyclonal anti–Hsp27 (1∶500)) diluted in PBS-T containing 0.5% fat-free milk powder. After three washes with PBS-T, membrane was incubated for 1 h at room temperature under gentle shacking with a horseradish peroxidase–conjugated secondary goat anti-rabbit IgG (1∶1000 dilution) in PBS-T containing 0.5% fat-free milk powder. The expected bands were visualized after incubation by enzyme-linked chemiluminescence for 5 min and quantified by scanning laser densitometry, normalizing to total amounts of the corresponding proteins.

### siRNA transfection

H9c2 cells at 3.5×10^3^ cells/cm^2^ were plated on 100 mm Petri dishes in media without antibiotics overnight before siRNA administration. Transfection of siRNA was completed following the protocol of the Lipofectamine 2000 manufacturer. The cells were placed in a 5% CO_2_ incubator at 37°C and 8 hrs after siRNA administration the media was replaced with fresh media without antibiotics. After 48 hrs total elapsed time, the cells were treated with FL3 (100 nM) for 10 h and then were treated with doxorubicin for an additional 14 h. Hsp27 siRNA (Cat #54502) and a negative control siRNA (Cat #4611) were purchased from Ambion [17]. The working concentration of siRNA in cell experiments was 30 nM. Data not shown: expression of Hsp27 in H9c2 cells transfected with siRNA for Hsp27 was reduced by 73%. Note that the expression of Hsp27 was not altered in H9c2 cells transfected with scrambling control siRNA.

### Animals

Male BALB/cByJ mice at 10 weeks (25–30 g) were divided into 5 groups. At day 0, doxorubicin or FL3+doxorubicin group received intraperitoneally (i.p.) a single dose of doxorubicin (Sigma) dissolved in 0.9% NaCl at 15 mg/kg [18]. At day −3, −2, −1, 0 and +3, FL3+doxorubicin, or FL3 group received (i.p.) a single dose of FL3 (0.1 mg/kg diluted in 20% of PEG 400). The vehicle groups received (i.p.) 20% PEG 400 diluted in water for the indicated date similar to FL3 group. Animal viability and body weight was recorded daily for 10 days.

### Histopathology and fibrosis

On day 10, after measurement of body weight, mice were killed. The heart tissue was fixed and 5 µm-thick paraffin sections were stained with Mallory tetrachrome staining for histological analysis [19]. Quantification of the fibrosis (blue staining) was performed as total pixel density of 20 individual, randomly selected fields from 10 different heart sections (n = 2 for each group), using NIH ImageJ.

### Detection of apoptosis

TUNEL analysis of fragmented DNA was performed according to the protocol of the manufacturer (Chemicon) as previously described [20]. Cells were pretreated with different concentrations of FL3 in serum free conditions, and then doxorubicin (1 µM) or its vehicle were added to the medium. A maximum and stable number of apoptotic cells were obtained within 24 h as a chronic model of cardiotoxicity. Cells were then fixed in 4% formaldehyde and permeabilized. After being washed, slides were incubated with TdT terminal transferase and fluorescein-dUTP. Slides were counterstained with DAPI. Cells were scored for TUNEL-positive nuclei corresponding to condensed DAPI stained nucleus. The percentage of TUNEL-positive cells was evaluated by viewing each field at ×40 magnification. Generally, 10 different microscopic fields containing 10–15 cells each were recorded for each sample. Each experiment was repeated at least three times. Cryosectioned mice heart samples were obtained 10 days after the treatments and then TUNEL assay was performed following the manufacturer's protocol. Slides were counterstained with DAPI and then were analyzed by fluorescence microscopy followed by quantification of the FITC total pixel density of 20 individual randomly selected fields from 10 different heart sections (n = 2 for each group), using NIH ImageJ.

### Analysis of Gene expression by RT-PCR

Total RNA from adult mice hearts was isolated using TRI®Reagent (Molecular research Center) and treated with DNase using the RNase-Free DNase Set. Semi-quantitative RT-PCR was performed on 0.5–5 µg of total RNA extracted from all hearts, using GAPDH as an internal control. The primers are shown in Supplementary Table S1.

### Statistic analysis

Data are expressed as mean ± SEM. Multigroup comparisons were performed using one-way ANOVA with post hoc correction. Comparisons between two groups were made using unpaired Student's t test. For all analyses, P<0.05 was considered significant utilizing Prism.

## Results

### Flavaglines protect cardiomyocytes against doxorubicin toxicity

**Figure 1:**
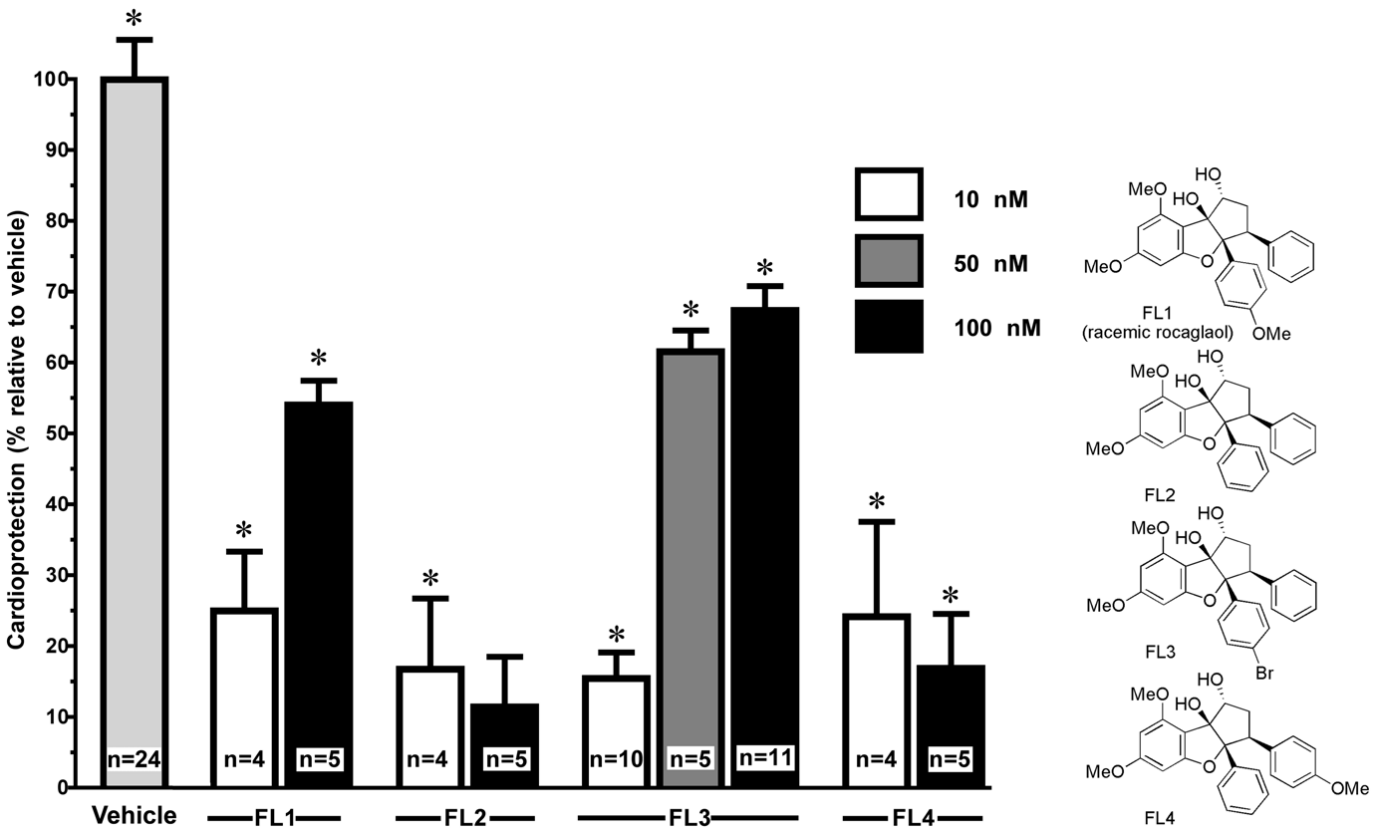
Flavaglines FL1–4 protect H9c2 cardiomyocytes from apoptosis induced by 1 µM doxorubicin. H9c2 cells were pretreated with flavaglines FL1–4 or their vehicle for 10 h and then treated with doxorubicin for additional 14 h (acute model of cardiotoxicity). Survival rate was quantified by FACS analysis. Histogram shows total population of surviving cells after different concentration of flavagline and doxorubicin treatments. Remarkably, incubation of H9c2 cells with 50 or 100 nM FL3 induced a cardioprotection of 61 and 67% respectively. The structure of the tested compounds is shown in the right panel. * indicates p<0.01 (n = 4).

The cellular activity of flavaglines was assessed using rat H9c2 cardiomyoblast cell lines that represent an established model to study doxorubicin-induced cardiotoxicity *in vitro* [21]. Excitingly, preincubation of H9c2 cells for 10 h with different concentrations of flavaglines significantly promoted cardioprotection against acute doxorubicin-induced apoptosis (Fig. 1, *P<0.01). None of these compounds alone had any perceptible proapoptotic effects (data not shown). The rank of cardioprotection by flavaglines in H9c2 cardiomyocytes was: FL2≈FL4≪FL1<FL3 (Figure 1). The most active compound, FL3, displayed maximum cardiomyocyte protection against doxorubicin-induced apoptosis by 61±3 and 67±3% at 50 and 100 nM concentrations respectively. Thus we concentrated on FL3 for the rest of our study.

**Figure 2:**
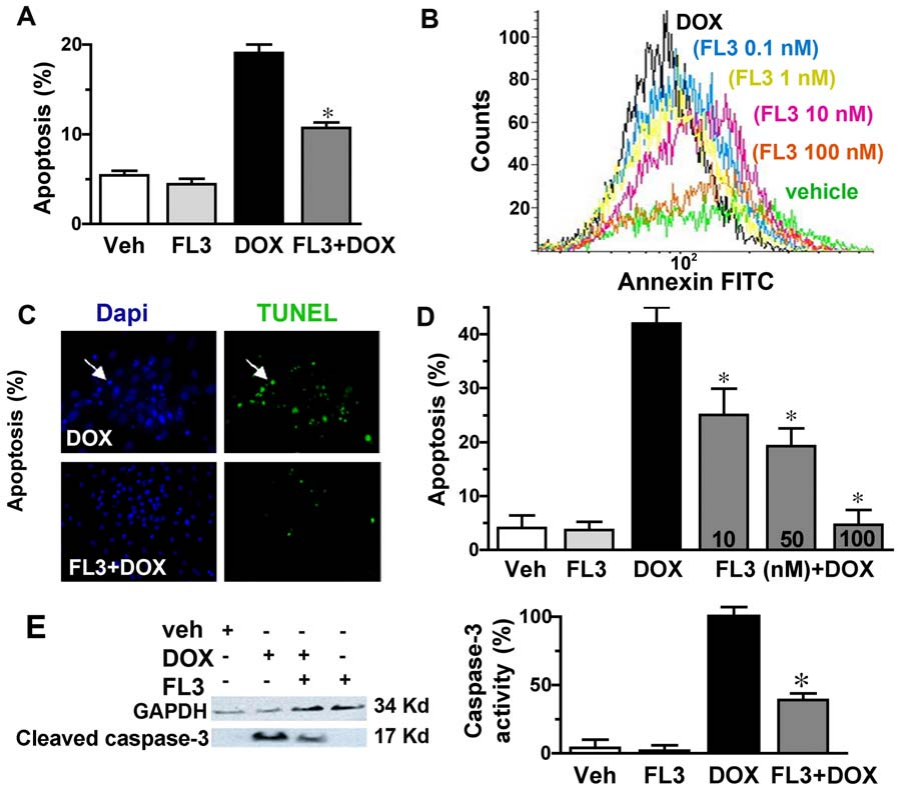
Protection of H9c2 cardiomyocytes by FL3 against doxorubicin-induced apoptosis. **A.** Histogram shows % of apoptotic cells in vehicle, FL3 (100 nM), doxorubicin (1 µM) and FL3+doxorubicin treated H9c2 cells detected by FACS analyses (n = 4, P<0.01). **B.** Original illustration of total apoptotic cells detected by FACS analyses among the cell population pretreated with FL3 (0–100 nM) and treated with doxorubicin (1 µM). **C.** Representative illustration of TUNEL positive apoptotic cells (green) versus Dapi (blue) positive total cells detected by fluorescent microscopic analyses for doxorubicin (DOX) and FL3+ DOX treated cells (n = 3, P<0.001). **D.** Histogram shows quantification of % of apoptosis (TUNEL positive cells versus Dapi positive total number of cells) for different concentration of FL3 (10–100 nM) in a chronic model of cardiotoxicity induced by doxorubicin (1 µM) (n = 3, P<0.001). **E.** Representative illustration of Western blot analyses on vehicle, doxorubicin, FL3 itself and FL3+doxorubicin treated cells, using active caspase-3 antibody and GAPDH antibody to normalize the assay. Quantitative analysis of Western blots is shown in histogram as % of caspase-3 activity over the vehicle (n = 3, P<0.01).

Next, we examined whether FL3 protects cardiomyocytes against an acute as well as a chronic treatment of doxorubicin. Figure 2A shows that incubation of the cells with 1 µM doxorubicin for 14 h (acute model) induced 22.7±0.8% apoptosis detected by FACS analysis utilizing Annexin V and PI. Pretreatment of the H9c2 cells with FL3 at 100 nM drastically reduced the proportion of apoptotic cell populations (Figure 2A, n = 4, *P<0.01). Figure 2B shows an original illustration of total apoptotic cell populations detected by FACS analyses. FL3 significantly reduced total apoptotic cell numbers induced by doxorubicin, in a dose dependent manner. The effect of FL3 was also examined in a chronic model of doxorubicin-mediated apoptosis by TUNEL staining. Indeed, FL3 significantly reduced apoptosis induced by a 24 h treatment of H9c2 cells with doxorubicin (1 µM), in a dose dependent manner (Figure 2C and 2D, n = 3, *P<0.001). The same results was observed when H9c2 cardiomyocytes where grown in a complete medium (Figure S1). Next, we investigated the effect of FL3 on activity of caspase-3, a downstream effector of apoptosis, using Western blot analysis with an antibody specific for the active (cleaved) form of caspase-3. Pretreatment by FL3 (100 nM) induced a 63% reduction in caspase-3 activity as compared to doxorubicin treatment alone, clearly confirming a cardioprotective effect of FL3 in H9c2 cells (Fig. 2E, n = 3, *P<0.01).

### FL3 protects cardiomyocytes against serum starvation

**Figure 3:**
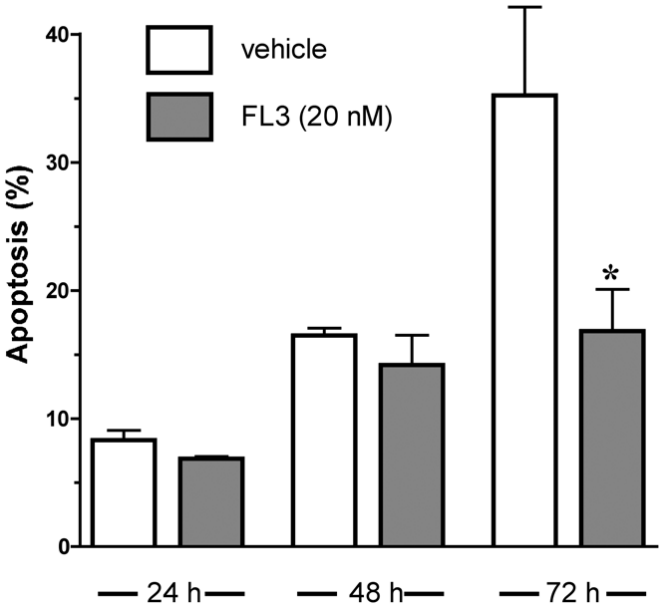
FL3 protects H9c2 cardiomyocytes against serum starvation. Histogram shows quantification of percentage of apoptotic cells in H9c2 cells that were cultured in 1% serum in presence of vehicle or FL3 (20 nM) for different time periods as detected by FACS analyses (n = 3, P<0.01).

To further explore the scope of flavagline cardioprotection, we examined whether FL3 could also protect H9c2 cardiomyocytes against serum starvation-mediated apoptosis. H9c2 cells were incubated with or without FL3 (20 nM) in the presence of 1% serum for 24 h, 48 h and 72 h and apoptosis was detected by FACS analyses. Maximum apoptosis was detected 72 h after serum withdrawal. Remarkably, in accordance with its protective efficacy against doxorubicin-induced apoptosis, FL3 diminished apoptosis by 50%, 72 h after serum starvation (Figure 3, n = 3, *P<0.01).

### FL3 cardioprotection is mediated through HSP27 phosphorylation

**Figure 4:**
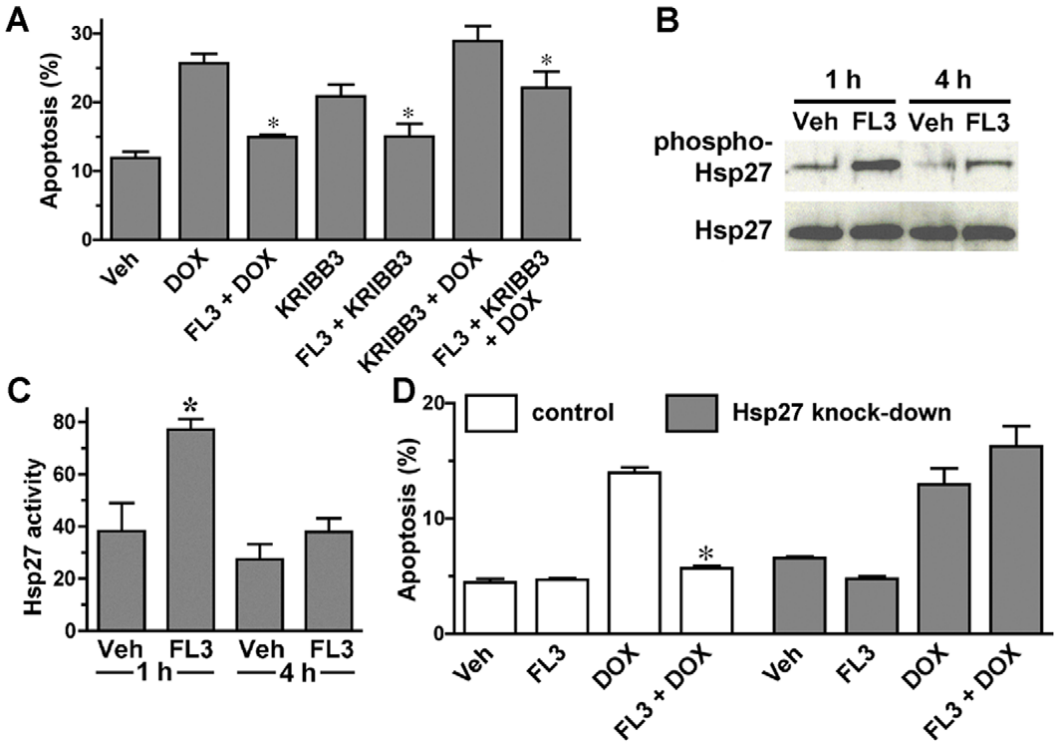
FL3 protects H9c2 cardiomyocytes by inducing Hsp27 phosphorylation. **A.** Quantification of the FACS analysis on H9c2 cardiomyocytes that were treated with doxorubicin (1 µM), FL3 (100 nM) in the presence or absence of Hsp27 inhibitor KRIBB3 (1 µM). **B.** Representative Western blot analysis on H9c2 cell lysates treated with vehicle or FL3 (100 nM) for 1 h or 4 h, utilizing antibody that recognize either phosphorylated (Ser-15 ) form of Hsp27 (upper) and total Hsp27 protein (lower bands). **C.** Quantitative analysis of the Western blots (% phosphorylated Hsp27 over total Hsp27 (n = 3, p<0.05). **D.** Histogram shows % of apoptotic cells on H9c2 control or H9c2 cells where Hsp27 expression was reduced by transfection of siRNA for Hsp27. Remarkably, in the H9c2 cells-knockdown for Hsp27, the cardioprotective effect of FL3 (100 nM) was completely reversed without modifying basal and doxorubicin-induced apoptosis as compare to H9c2 control cells (n = 3, p<0.05).

To examine the signaling mechanism involved in FL3-mediated cardioprotection, pharmacological inhibitors of pro-survival kinases such as ERK-1 and -2, STAT3, p38 MAPK and Akt were utilized (PD98059, WP1066, SB235090 and LY204002 respectively). None of these inhibitors significantly blocked the cardioprotective effect of FL3 on H9c2 cardiomyocytes treated with doxorubicin (data not shown). Since phosphorylation of a small heat shock protein Hsp27 induces a cascade of events that protect cardiomyocytes from many stresses [22]–[25], we examined whether this protein is involved in FL3-survival signaling pathway. Interestingly, KRIBB3, an inhibitor of Hsp27 [26], completely reversed FL3-mediated cardioprotective effect (Fig. 4A). Since inhibition of the Hsp27 compromised the cardioprotective effects of FL3, the ability of FL3 to activate this pathway was evaluated and quantified by Western blot analysis and densitometry, using antibodies specific for Hsp27 phosphorylated on Serine 15. Figure 4B illustrates original Western blot analyses that FL3 induces Hsp27 phosphorylation, reaching to maximum level within 1 h without altering the level of total Hsp27 protein as quantified and shown in Figure 4C (n = 3, *P*<0.05). Involvement of Hsp27 in the cytoprotective effect of FL3 was further confirmed by knocking-down Hsp27. As shown in Figure 4D, FL3-mediated protection of H9c2 cells against apoptosis induced by doxorubicin was completely reversed in H9c2 cells where Hsp27 expression was knocked-down as compared to FL3 effect on control H9c2 cells.

### FL3 protects against doxorubicin-induced cardiotoxicity *in vivo*

**Figure 5:**
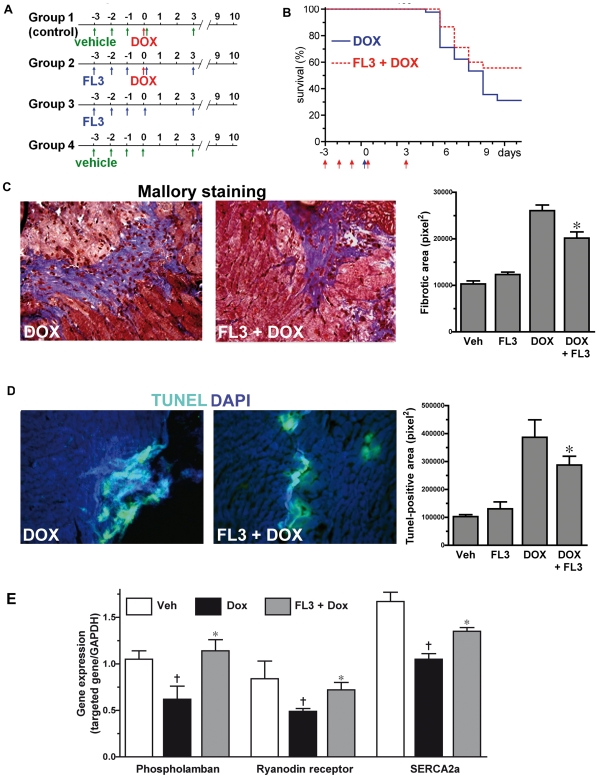
*In vivo* cardioprotective activity of FL3 against doxorubicin -induced apoptosis and fibrosis. **A.** Protocol of intra peritoneal administration of compounds in mice model of doxorubicin-induced cardiotoxicity. **B.** Kaplan-Meier survival curves for mice (n = 45) treated with either doxorubicin (15 mg/kg i.p.) or doxorubicin +5 injections of FL3 (12.5 µg/kg i.p.). The survival rate significantly increased in the FL3+doxorubicin group (n = 45) compared with doxorubicin-treated animals (56 versus 31%, p = 0.024). There was no mortality in the control group and FL3 group. **C.** *In vivo* myocardium fibrosis. Representative illustration of Malory tetrachrome staining for doxorubicin (DOX) and FL3+DOX treated mice hearts, demonstrating different degrees of fibrosis (blue) in their hearts. The histogram shows the quantification of fibrotic areas (density/pixels^2^) in the cryosectioned hearts of 4 groups of mice. **D.** *In vivo* myocardial apoptosis. Representative TUNEL positive (green) apoptotic area in heart tissue sections from animals treated with DOX and FL3+DOX, original magnification is ×20. Dapi is a nucleus marker (blue). The FL3+doxorubicin groups had fewer apoptotic nuclei compared with the doxorubicin group. The plots show the quantification of TUNEL positive apoptotic area (density/pixels^2^) in the cryosectioned hearts of 4 groups of mice (n = 4, P<0.001). **E.** Quantitative RT PCR analyses of cardiomyocyte contractile genes phospholamban, ryanodin receptor and SERCA2a. **†** indicates p<0.05 as compare to vehicle, * indicates p<0.05 as compare to doxorubicin group.

To further explore the cardioprotective effect of FL3 *in vivo*, 5 groups of mice received different treatments, according to the protocol shown in Figure 5A. At day 12, doxorubicin-treated animals had a compromised survival rate (31%; n = 45), and there was a remarkable increase in survival rate in mice treated with FL3+doxorubicin (56%; n = 36) (P = 0.024, corrected Fisher's exact P = 0.04)” (Figure 5B). The toxicity of doxorubicin was also manifested by a 22% loss of body weight at day 4 (n = 44). However, mice pretreated with FL3 exhibited only a 13% (n = 45, p<0.01) loss of body weight. Mice treated with doxorubicin displayed also a marked reduction in heart weight at day 4 that was significantly alleviated by the pretreatment with FL3 (Supplementary Figure S2). Note that the survival rates of animals in the control group and FL3 alone group were consistently 100% in all series of experiments. This data indicates also that FL3 displays a proper bioavailability.

We next analyzed morphological changes in all surviving mice at the 12 days. The myocardial pathology associated with doxorubicin treatment, including myocyte loss and fibrosis. Representative staining of heart sections by Mallory tetrachrome can be seen in Figure 5C for doxorubicin and FL3+doxorubicin treated hearts, demonstrating significantly less fibrotic tissue (blue) in FL3+ doxorubicin treated group. The fibrotic area was reduced in the FL3+doxorubicin treated group as compared with doxorubicin treated groups (Figure 5D, histogram, n = 4, *P<0.001). No cardiomyocyte pathology and fibrosis were detected in the vehicle- treated control or FL3 alone-treated groups. Quantitative RT-PCR analyses demonstrated that FL3 reversed the increase of expression of collagen 2a1 induced by doxorubicin, confirming the antifibrotic activity of FL3 (Supplementary Figure S3). Representative TUNEL (green) stained heart sections are shown in Figure 5D, demonstrating fewer apoptotic cells in the FL3-treated mouse heart. Quantitative analyses by TUNEL assay on the heart sections revealed a significant decrease in cell death in FL3+doxorubicin treated hearts as compared to doxorubicin alone treated hearts (n = 3, *P<0.05, Figure 5D, histogram). This is consistent with the *in vitro* cardioprotective effect of FL3 in H9c2 cells (Figure 2C). RT-PCR analyses revealed that FL3 treatment prevented doxorubicin-induced decreased expression of connexin 43 (Supplementary Figure S3), a cardiomyocyte gap junction channel gene that is involved in cellular communication and mitochondrial apoptosis [27], [28]. All together these findings can account for the ability of FL3 to attenuate the deleterious effect of doxorubicin on fibrosis, apoptosis and intercardiomyocyte communication.

To further examine whether these beneficial effects of FL3 contribute to its protection against the adverse effects of doxorubicin on the contractions of cardiomyocytes, the expressions of intracellular Ca^2+^ handling genes as indicators of changes in cardiomyocyte contractility were assessed. In agreement with the previous studies, doxorubicin-treated mice hearts exhibited reduced level of Ca^2+^ handling genes such as the sarcoplasmic/endoplasmic reticulum Ca^2+^ ATPases (SERCA), phospholamban and the ryanodin receptor. FL3 treatment prevented doxorubicin-induced decreased expression of SERCA2a, phospholamban and the ryanodin receptor (Figure 5E). Consistent with the ability of FL3 to attenuate the adverse effects of doxorubicin on the development of fibrosis and cell death, FL3 also prevented the decline in calcium handling gene expression, thereby improving cardiac contractility.

## Discussion

This study is the first attempt to characterize the beneficial effect of a flavagline in doxorubicin-induced cardiac damage. Cardiotoxicity represents the most common reason for termination of the use of anthracyclines in cancer chemotherapy. The search for cardioprotective agents that alleviate anthracyclines cardiotoxicity in the last 30 years has led only to dexrazoxane [29]. This iron chelator is the only cardioprotective agents with proven efficacy in cancer patients receiving anthracycline treatment. However, its effectiveness is limited, and even not established in children [30]. Although a number of alternative cardioprotective strategies have been assessed in animal models, none of them have been clinically validated yet [31], [32].

The unique biological profile of flavaglines has attracted considerable interest these last ten years, [8], [33]. Although neuroprotective effects of flavaglines have been described [9], our data unravel for the first time the potential of flavaglines in the prevention of anthracycline cardiotoxicity. As shown in Figure 1, FL3 displayed a stronger cardioprotection that FL1, which is the racemate of the natural compound rocaglaol, indicating a preference for a bromine over a methoxy in the phenyl moiety in position 5. Deletion of this methoxy or its displacement to the adjacent phenyl moiety was greatly detrimental for cardioprotection.

The cardioprotective action of flavaglines was not restricted to damages induced by doxorubicin in the cultured cardiomyocytes. FL3 strongly reduced also the apoptosis induced by serum starvation. Interestingly these stresses are of different nature: doxorubicin induces an oxidative stress, while serum starvation blocks growth factors signaling that is necessary to cell survival, mimicking an important component of myocardial ischemia.

Our study presents the first evidence that flavaglines display their cytoprotective effect by inducing the phosphorylation of Hsp27. This small heat shock protein has recently emerged as a critical factor for the protection of cells against many insults, through a myriad of functions including chaperone activity, mRNA stabilization, maintenance of cytoskeletal architecture, control of redox homeostasis, and inhibition of apoptosis [22]. Consistent with our findings, overexpression of Hsp27 and hyperthermia-induced phosphorylation of Hsp27 were shown to strongly protect cardiomyocytes against doxorubicin insult [25], [34]. The identification of the molecular target of flavaglines and the detailed molecular mechanism of Hsp27 activation underlying flavagline cardioprotection are currently under investigation.

Cardioprotective effect of FL3 was demonstrated in an *in vivo* model as well. A single dose of doxorubicin induced a severe myocyte loss due to apoptosis and fibrosis in heart, which lead to a loss of heart weight and a mortality rate of 69%. This loss of heart weight is characteristic of this model of acute cardiotoxicity following a single injection of doxorubicin in adult mice [35]–[37] (while models of chronic doxorubicine-induced cardiotoxicity generally lead to an increase of heart weight due to cardiac hypertrophy). Agreement with the protective efficacy of FL3 in H9c2 cardioblasts, treatment of mice with FL3, prevented doxorubicin-induced apoptosis and fibrosis in heart and reduced mortality to 44%. Together with the *in vitro* studies showing that FL3 signaling produces a direct anti-apoptotic effect in cultured H9c2 cardioblasts, our *in vivo* results suggest that prevention of apoptosis is a possible mechanism of FL3-mediated cardioprotective effects against the effects of doxorubicin. Even though we did not directly compare the effect of dexrazoxane in our study, based on previous reports, it appears that FL3 is as effective as this clinically used cardioprotective agent in mice model [38]. Our previous *in vivo* study showed that FL3 alone does not induce any loss of weight or any other sign of toxicity in mice [14].

Previous studies showed that doxorubicin decreased calcium handling gene expression in cultured new born [39], adult rat ventricular cardiomyocytes [40], and rabbit *in vivo* model of doxorubicin cardiotoxicity [41], [42], leading to myocardial dysfunction [28]. In concert with the previous studies, here we illustrated that doxorubicin treated mice hearts exhibited reduced level of these Ca^2+^ handling genes, as indicators of a sarcoplasmic and contractility dysfunction. FL3 treatment prevented attenuation of these genes, indicating that FL3 significantly alleviated these signs of cardiac dysfunction induced by doxorubicin.

Taken together, reduced heart weight and contractile gene expression, increased apoptosis and fibrosis, are the principal signs of the cardiotoxicity of doxorubicin. Combining our *in vitro* in the cardiomyocytes and *in vitro* in mice, our data clearly demonstrated that FL3 treatment attenuated these adverse effects of doxorubicin and significantly increased the survival rate *in vivo*.

Collectively, our *in vitro* and *in vivo* data provide the first evidence of the cardioprotective effects of FL3 against the adverse effects of doxorubicin on the structure and function of heart.

Flavaglines exhibit not only cardioprotective, but also neuroprotective effects. In addition to their anticancer and cardioprotective activities, Bayer scientist documented the in *vivo* neuroprotective effects of flavaglines in animal models of stroke and Parkinson disease [9]. Interestingly, recently Pelletier and collaborators showed that flavaglines enhance doxorubicin chemosensitivity in a mouse lymphoma model [43]. Albeit this profile of pharmacological activities of flavaglines is original, it is not unique: tetracyclines, which are broad-spectrum antibiotics, are currently undergoing clinical trials not only for several types of cancers, but also for neurological and cardiovascular diseases (Huntington, Parkinson and Alzheimer's diseases, coronary artery bypass and post-myocardial infarction remodeling) [44]. A tetracycline was shown to alleviate also doxorubicin-induced cardiotoxicity in mice [45]. Similarly, histone deacetylase inhibitors, which were originally developed to treat cancers, have emerged as promising drug candidates for the treatment of heart failure and neurodegenerative disorders [46]. The benefit of an association of flavaglines to anthracyclines might be double: these compounds could enhance the anticancer effects of anthracyclines [14] and alleviate their main adverse effect, cardiotoxicity. Considering that anthracyclines are the most widely used anticancer drug, the discovery of a new class of cardioprotective agents is of paramount importance in clinic, and further studies to validate these hypotheses are warranted. The results of this study raise also the possibility that flavaglines may protect other organs than heart against the damaging effects of cancer chemotherapies, suggesting that these compounds deserve further extensive preclinical investigations.

## Supporting Information

Figure S1 **Protection of H9c2 cardiomyocytes grown in a complete medium (10% serum) by FL3 against doxorubicin-induced apoptosis.** Histogram shows % of apoptotic cells in vehicle, FL3 (100 nM), doxorubicin (1 µM) and FL3+doxorubicin treated H9c2 cells detected by FACS analyses (n = 5, p<0.05).
(TIF)

Figure S2 **The heart weight as an indirect sign of cardiomyopathy in mice treated with doxorubicin and/or FL3.** Histogram shows the heart weight (mg) of mice treated with vehicle, FL3 (5 injections at 12.5 µg/kg i.p.), doxorubicin (15 mg/kg i.p.) or doxorubicin+FL3 according to the general protocol displayed in Figure 5A. Mice were euthanized 4 days after the administration of doxorubicin or vehicle and hearts were weighted.
(TIF)

Figure S3 ***In vivo*** **cardioprotective activity of FL3 against doxorubicin-induced modification of fibrotic and cardiomyocyte structural gene expression.** Quantitative RT PCR analyses for collagen 1a1, collagen 2a1 and connexion 43 were performed on RNA extracted from hearts of mice treated with vehicle, doxorubicin or doxorubicin+FL3 according to the general protocol displayed in Figure 5A. Mice were euthanized 4 days after the administration of doxorubicin or vehicle and hearts were taken off for RNA extraction. * indicates p<0.05 as compare to vehicle, **†** indicates p<0.05 as compare to doxorubicin group.
(TIF)

Table S1 **Primers used to analyze gene expression by RT-PCR.**
(DOC)
