# Supplementary figures and images for: Flavaglines Alleviate Doxorubicin Cardiotoxicity: Implication of Hsp27
Source: PLoS One. 2011 Oct 31;6(10):e25302. doi: 10.1371/journal.pone.0025302 (PMC3204970; doi:10.1371/journal.pone.0025302)

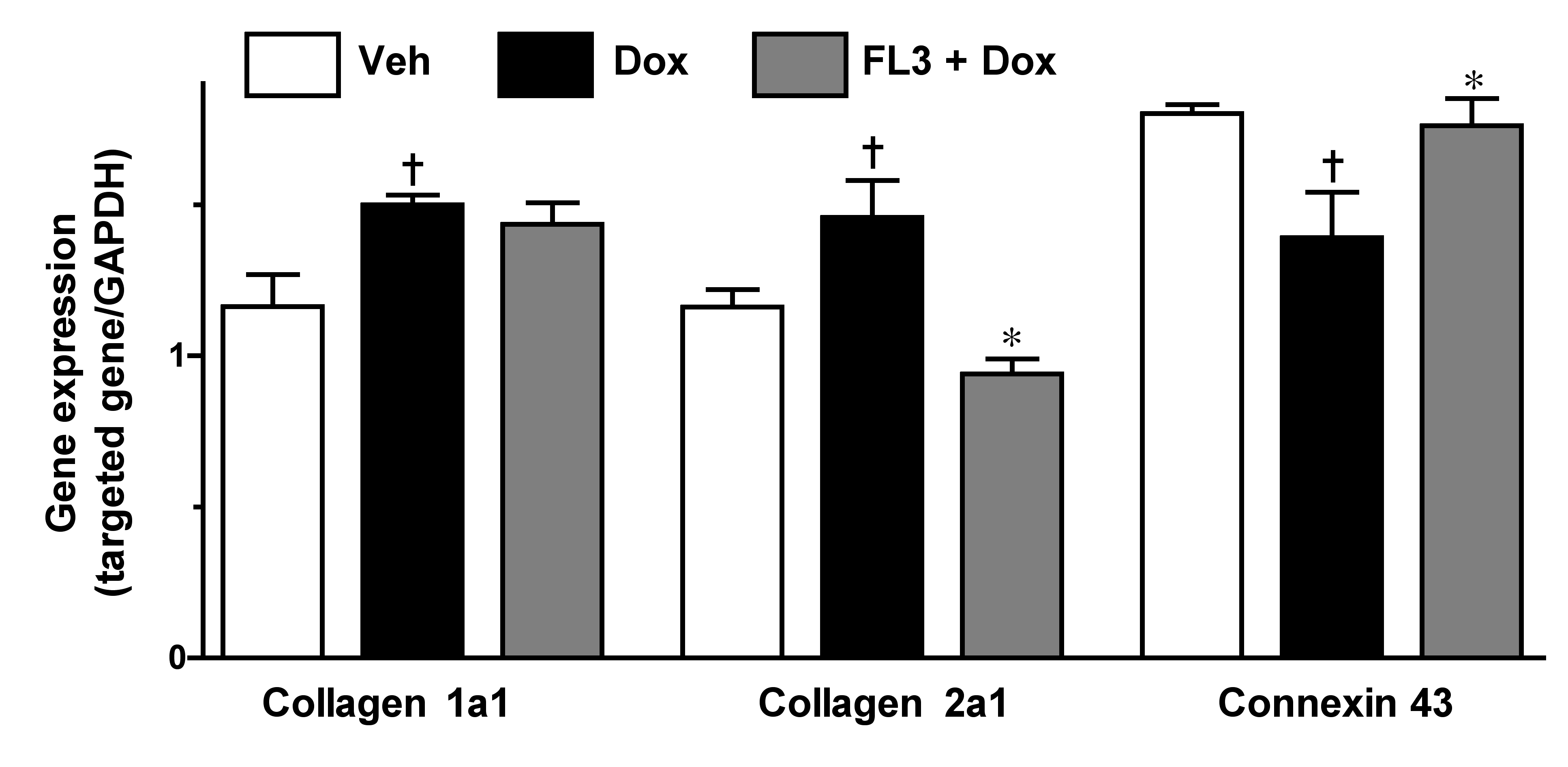

Supplement: Figure S3 — In vivo cardioprotective activity of FL3 against doxorubicin-induced modification of fibrotic and cardiomyocyte structural gene expression. Quantitative RT PCR analyses for collagen 1a1, collagen 2a1 and connexion 43 were performed on RNA extracted from hearts of mice treated with vehicle, doxorubicin or doxorubicin+FL3 according to the general protocol displayed in Figure 5A. Mice were euthanized 4 days after the administration of doxorubicin or vehicle and hearts were taken off for RNA extraction. * indicates p<0.05 as compare to vehicle, † indicates p<0.05 as compare to doxorubicin group. (TIF) [file pone.0025302.s003.tif]
