# Supplementary material for: Flavaglines Alleviate Doxorubicin Cardiotoxicity: Implication of Hsp27
Source: PLoS One. 2011 Oct 31;6(10):e25302. doi: 10.1371/journal.pone.0025302 (PMC3204970; doi:10.1371/journal.pone.0025302)
Supplement: Table S1 — Primers used to analyze gene expression by RT-PCR. (DOC) [file pone.0025302.s004.doc]

**Supplementary Table S1:** primers used to analyze gene expression by RT-PCR

| Gene | Primer Sequence |
| --- | --- |
| Phospholamban | F 5'-TCT CCC TAC TTT TGC CTT CCT G-3' |
|  | R 5'-TCT CAC AAA GCT GTT CTC AGC-3' |
| Ryanodin receptor | F 5'- GAA TCA GTG AGT TAC TGG GCA-3'- |
|  | R 5'- CTG GTC TGA GTT CTC CAA AAG-3' |
| Serca 2a | F 5'-CAA TAC TGG AGT AAC CGC-3' |
|  | R 5'-AAC TTC TCT GGA GAG GC-3' |
| Serca 2b | F 5'-GTA AAG AGT GTG TGC AG-3' |
|  | R 5'-GAA ACC TCC TTC ACC AGC-3' |
| Col 1a1 | F 5'-TGT CCC AAC CCC CAA AGA C-3' |
|  | R 5'-TCG GTG TCC CTT CAT TCC-5' |
| Col 1a2 | F 5'-GAA CGG TCC ACG ATT GCA TG-3' |
|  | R 5'-CTT GCA GGA CCC GTT TGA CC-3' |
| Connexin-43 | F 5'-GCC GGC TTC ACT TTC ATT AAG-3' |
|  | R 5'-GCC ACC TCT CAT CTT CAC CTT G-3' |
